# Institutional Resistance to Medical Assistance in Dying in Canada: Arguments and Realities Emerging in the Public Domain

**DOI:** 10.3390/healthcare11162305

**Published:** 2023-08-15

**Authors:** Michelle Knox, Adrian Wagg

**Affiliations:** Department of Medicine, University of Alberta, Edmonton, AB T6G 2P4, Canada

**Keywords:** medical assistance in dying, assisted dying, euthanasia, right to die, conscientious objection, conscientious refusal to treat, palliative care

## Abstract

Since the legalization of medical assistance in dying (MAiD) in Canada in 2016, volitional non-participation in MAiD on the part of some healthcare institutions has revealed ethical uncertainties, potential access problems, and policy gaps. The problem has remained much neglected in the literature base, with no comprehensive studies on the subject so far. We analyzed print media articles and grey literature on institutional objections to and non-participation in MAiD. Thematic analyses were performed on all data to better understand the diverse stakeholder arguments and positions that characterize this important public health debate. Our search yielded 89 relevant media articles and 22 legislative, policy, and other relevant documents published since 2016 in the English language. We identified four main themes about institutional refusals to participate in MAiD, articulated as the following questions: (1) Who has the right to conscience? (2) Can MAiD be considered a palliative practice? (3) Are there imbalances across diverse stakeholder rights and burdens? and (4) Where are the gaps being felt in MAiD service implementation? Stakeholder views about institutional conscience with respect to MAiD are varied, complex, and evolving. In the absence of substantial systematic evidence, public domain materials constitute a key resource for understanding the implications for service access and determining the relevance of this contentious issue for future MAiD research and policy.

## 1. Introduction

On 17 June 2016, Bill C-14 came into force and effect, legalizing medical assistance in dying (MAiD) in Canada. Since then, a number of challenges to legislative interpretation and service implementation have emerged [1,2,3,4]. Among these, healthcare institutions that choose not to participate in MAiD present a complex area of concern [5,6,7,8,9], with ethical, social, and medicolegal tensions between collectively held conscience rights and patient rights to equitable healthcare access [10,11]. While several other MAiD-related challenges have emerged as top research priorities, institutional resistance to MAiD—and the resultant impacts on patients—has remained a largely uninvestigated, but real and important problem.

Historically, much of the discourse around the legalization of assisted dying, in Canada and elsewhere, has been driven by the goal of social justice, with autonomy and dignity presented as core guiding concepts to appeal for legislative change [12]. As per the Supreme Court of Canada’s rulings in court cases preceding Bill C-14 (Canadian federal legislation to outline the requirements for the MAiD provision), “an individual’s response to a grievous and irremediable medical condition is a matter critical to their dignity and autonomy” [13] (para 66). Likewise, arguments favouring MAiD—spanning legal, social, and political views—have consistently framed the right to die as a natural extension of the right to life, liberty, and security of person [14,15] and, therefore, as an essential human right. The Supreme Court ruling legalizing MAiD reflects these ideas, with the intent to uphold personal liberty and security of person, as guaranteed under the Canadian Charter of Rights and Freedoms [16]. Therefore, at present, MAiD is fully integrated within Canada’s socialized healthcare system and widely conceived as a medical service every eligible Canadian may avail of.

Conversely, however, it has also been argued that there is no explicitly articulated right to healthcare access within the Canadian constitution, Charter or otherwise, nor the right to any particular healthcare service [17]. Yet, a right to healthcare—and therefore, to public healthcare services—can be inferred from the Charter protections set out under section 7 (right to life, liberty, and security of person) [16]. At the same time, the Canadian Charter clearly and explicitly protects individuals from having to perform, as part of their employment duties, any task that is against their moral or religious beliefs; that is, the right to conscientious objection [18]. Based on this stipulation, some publicly funded healthcare institutions—primarily those with religious affiliations—have declined to provide MAiD services on their premises. Given that a large proportion of end-of-life care (EoLC) and palliative care services in Canada are provided by religiously affiliated institutions, this raises fundamental questions about potential effects on equitable and well-integrated access to MAiD within the health system [19].

To our knowledge, no systematic studies to date have evaluated the nature and scope of this problem and its possible ramifications across the EoLC service landscape. In part, the paucity of research on this sensitive topic may stem from general unclarity regarding the number of non-participating sites in the country and whether prohibitions at every such site prevent only the administration of lethal procedures or pose hindrances to MAiD assessments as well. There is also little known about the number of individuals who choose, where possible, a healthcare facility that aligns with their views on MAiD, how many individuals re-route their MAiD requests from one site to another (including to their own homes), and ultimately, how many are unable to receive MAiD due to institutional/structural factors.

Given the geographic variability of MAiD service organization across the country [20,21] and the consequent challenge of systematizing such an investigation, we suggest an alternate focus to set the groundwork for orienting further inquiry. We thus offer, in this paper, an analysis of stakeholder perspectives, experiences, and positions—as available from public domain sources—to explore the contours of the dilemma, its relevance for MAiD integration in the Canadian public health system, and its worthiness for further research and policy reform. Despite its importance from a bioethical standpoint, most knowledge about the subject is presently constructed through media sources, advocacy and organizational platforms, and public health information channels. Therefore, utilizing media articles and grey literature as our primary data sources, we identify the key arguments articulated by diverse stakeholders about institutional non-participation in MAiD.

## 2. Materials and Methods

### 2.1. Data Sources

**Figure 1:**
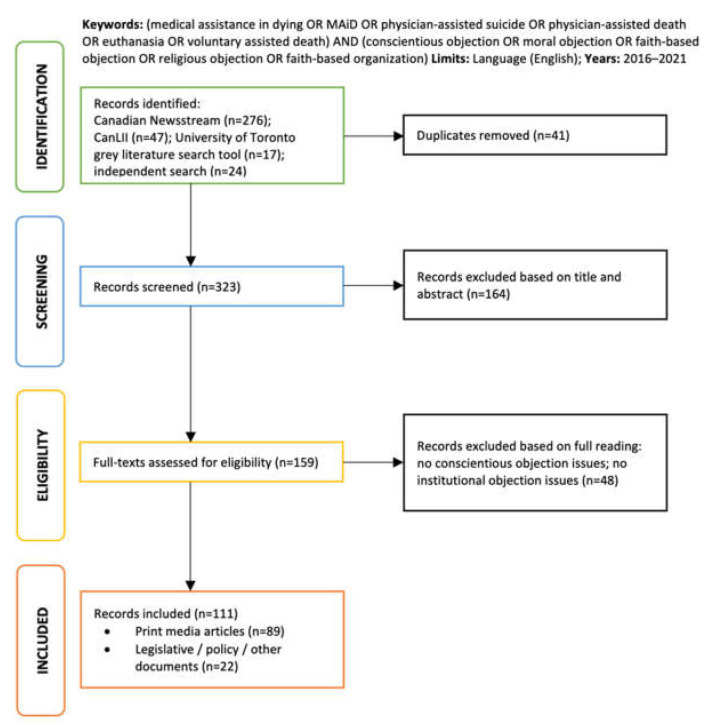
PRISMA diagram of search and selection.

We performed an exhaustive electronic search of 2 large databases, Canadian Newsstream and CanLII. Canadian Newsstream focuses exclusively on Canadian print media and includes over 350 news sources (i.e., national, provincial, and regional newspapers and magazines) in its database. CanLII is a database provided by the Canadian Legal Information Institute, where the full text of all court judgments, tribunal decisions, statutes, and policy regulations from all Canadian jurisdictions are available. It also provides a subsidiary database, CanLII Connects, where case commentaries, summaries, and opinion pieces from lawyers, scholars, and other legal experts can be found. A supplementary search, using a search tool developed by the University of Toronto Library, was performed to collect government documents (reports, policy papers, etc.). This was augmented by an independent scan of healthcare organization websites to capture relevant information (press releases, position statements) that would be otherwise difficult to locate in traditional databases. Our search terms included “medical assistance in dying” (and related terms, i.e., “MAiD”, “euthanasia”, “physician-assisted death”, “physician-assisted suicide”) AND “conscientious objection” (and related terms, e.g., “moral objection”, “faith-based objection”, “religious objection”, “faith-based organization”). Articles from non-Canadian jurisdictions were excluded to focus our analysis to the Canadian context. The complete search and elimination sequence is available in Figure 1.

### 2.2. Data Analysis

All data were uploaded to ATLAS.ti (v 9.0, Atlas.ti Scientific Software GmbH, Germany), a software program for computer-aided qualitative analysis. Based on a preliminary review of relevant field literature, some open-ended codes were determined and discussed in an initial meeting between the authors. In the first stage of reading, these initial codes (e.g., autonomy, conscience, patient rights) were used to organize, describe, and sort the data. Second, through an in-depth, analytical reading of each document, further codes were identified inductively from the dataset, and preliminary themes were generated from code groupings. At the third stage, each document was re-read, using emergent themes to guide and refine our analysis. The authors, MK and AW, met over the course of the analysis to discuss the interpretation of themes and to ensure that there had been no significant omissions. A final interpretation was then built with respect to the key argument themes that construct the public debate on institutional objections to MAiD.

## 3. Results

After redundant, duplicate, and unrelated items were discarded, our search yielded a dataset of 89 media articles and 22 legislative, policy-related, or public health information documents published in the English language after 2016, after the MAiD legislation came into effect and up until the present. Targeted media articles were regular and in-depth news reports, letters or comments to the editor, columns, editorials, and guest editorials. Our grey literature search included court challenges, internal policy regulations, and position statements issued by healthcare institutions, organizations, professional bodies, groups, and other collectives. We explore, in greater depth, the potential implications of stakeholder discourses on MAiD service accessibility for the Canadian health system elsewhere [22]. In this paper, we focus on describing the key themes of argumentation that underpin this important public health debate, highlighting the future research potential of this topic.

In the forthcoming sections, we refer to commentators in support of collective/institutional conscience rights as *proponents* and those against it as *opponents*. Our analysis identified four principal themes, which we frame as the following four questions: (1) Who has the right to conscience? (2) Can MAiD be considered a palliative practice? (3) Are there imbalances across diverse stakeholder rights and burdens? and (4) Where are the gaps being felt in MAiD service implementation? Categories of commentators were not mutually exclusive, with arguments and positions found to be inextricably interlinked. We now describe each of these in turn.

### 3.1. Who Has the Right to Conscience?

By and large, commentators favoured the rights of individual healthcare professionals to abstain from MAiD procedures for reasons of conscience; however, there was discord over whether individual conscience rights can—or should—be extended to healthcare-providing institutions. The voices dominating these discourses were primarily those of faith-based groups and non-participating institutions on the one hand (proponents), and legal/ethical experts, healthcare professionals, and patient advocacy organizations on the other (opponents). We provide some illustrative quotes in Table 1.

Proponents of collective conscience rights framed institutional non-participation in MAiD as a matter of religious freedom guaranteed by the Canadian Charter of Rights and Freedoms. Rejecting their position, opponents maintained that only people—and not collective entities—possess moral conscience, and by extension, only people may lay claim to legal protections. Since the Canadian Charter already protects individuals from having to perform professional duties that conflict with their religious/moral values, opponents maintained that no additional legislative safeguards to protect collective entities were needed [29,30]. However, adding further complexity to the dispute, proponents noted that healthcare institutions cannot be dissociated from their faith-driven founders, supporters, funders, or indeed, from the individuals who help operate and provide care at these sites today—often motivated by their religious calling and values [25].

Another justification offered in favour of institutional non-participation in MAiD was that all medical services are not necessarily available at every healthcare facility [25,31]. As such, proponents asserted that the non-universality of MAiD service provision—like other variably distributed medical services—is consistent with health system norms. In response, the counterview held that morally motivated refusals to provide medical services cannot be equated with service limitations resulting from infrastructural constraints or inadequate specialist expertise at a certain healthcare facility [28]. Some opponents also speculated that granting institutional exemptions from MAiD bears parallels to and could establish undesirable legal precedent in relation to other contentious medical services, many of which already involve access barriers or inequitable distribution challenges to begin with (e.g., abortion, LGBTQ health services, etc.) [29,32,33,34].

The most prominent opposing view within this theme was that taxpayer-funded healthcare institutions should not be granted blanket exemptions from the provision of any medical service to which the public has a legal right [26,27,33,35]. Some suggested that, if granted these exemptions, the costs of transferring patients to alternative healthcare facilities should be borne by MAiD-declining institutions, rather than by the health system/taxpayers [36]. In answer, proponents of collective conscience argued that public funding for healthcare also includes the tax contributions of those who do not support MAiD, and accordingly, these citizens’ values should not be ignored in determining the appropriate use of taxpayer funds [25]. They further noted that healthcare institutions receive, in addition to public funds, substantial financial support from private donors who align themselves with the religious mandates of the institutions to which they donate, with the expectation that these mandates will be upheld [25,37].

### 3.2. Can MAiD Be Considered a Palliative Practice?

There appeared to be considerable uncertainty about positioning MAiD within medical practice, and specifically, within palliative care. Elements within this theme were found to be entangled with longstanding tensions regarding medical paternalism, death denialism, and the politics of clinical overreach in (and control over) natural biological processes. Institutional conscience proponents within this theme comprised faith-based groups, members of the public, and some palliative care professionals. Opponents comprised patient advocacy organizations, lay citizens, and other palliative care professionals. We provide some illustrative quotes in Table 2.

One argument pertained to the meaning, role, and scope of palliative practice in relation to MAiD. Collective conscience proponents conceived palliative medicine as therapeutic care for improving the quality of life of individuals with terminal illnesses, and thus, situated MAiD outside the core palliative mission. Some highlighted the (faith-based) cardinal principle of the sanctity of life, and correspondingly, of medicine’s goal to defend life from harm, i.e., from death [44,45]. Accordingly, they portrayed MAiD as a consequence of a deficient health system or the palliative inexpertness of indifferent doctors who fail to provide compassionate psychological care at the end of life [39,46]. Meanwhile, collective conscience opponents considered MAiD to be well aligned with palliative care objectives, framing MAiD as one of many medical procedures to ameliorate suffering in accord with patient conceptions of dignity, autonomy, and informed decision-making at the end of life [34,47,48]. Moreover, many opponents also believed that honouring a MAiD request demonstrated compassion and respect for patient wishes and, therefore, signified a moral obligation on the part of healthcare professionals towards MAiD-seeking patients [43,49].

A related argument focused on the extent to which palliative care professions—and associated healthcare institutions—are driven by religious intent and agenda. Proponents of collective conscience emphasized that palliative medicine neither intentionally hastens nor prolongs death but aims to minimize the pain and discomfort of the dying, with no single religious or spiritual scheme driving its goals. Others disagreed, alleging that institutional stances against MAiD are shaped by a long history of the religious organization of palliative care services. Some opponents also pointed to the lingering influence of redemptive suffering concepts within Christian medicine, where end-of-life pain might be seen as a parallel for Christ’s agony, with the promise of resurrection after death [50]. Proponents dismissed these views, asserting that it is, instead, the Hippocratic oath that makes MAiD difficult to reconcile with medicine’s fundamental do-not-harm principle [51,52]. This claim was countered on the grounds that subjecting grievously ill patients to transfers does not align with do-no-harm principles [43].

In a more politically charged framing, federal MAiD legislation was interpreted by some as a national endorsement of pro-death ideology [38]. These worries were often assimilated into broader apprehensions about the modern normalization of suicide as a legitimate solution to human suffering and concerns around protecting vulnerable persons (e.g., older adults; dementia patients; people with disabilities; and more recently, people with medically manageable mental illnesses) from the risk of inducement to suicide [53]. Some proponents noted that MAiD requests decline when high quality palliative care is readily available, characterizing the desire to end one’s life as a failure of palliative medicine and, more generally, of public health systems where palliative care is not a well-integrated and universally accessible service [39,46,54]. This argument shifted the focus of institutional liability in inequitable MAiD access towards the need for ensuring its implied alternative, that is, appropriate and adequate palliative care provisions across the public health landscape.

### 3.3. Are There Imbalances across Diverse Stakeholder Rights and Burdens?

This theme related to the complex need and difficulty of achieving a point of reasonable compromise between the rights of conscientious objectors to abstain from—and the rights of patients to receive—MAiD services. Here, commentators were interested in identifying where the risk of harm was situated disproportionately. Given that institutional and individual interests appeared entwined here, we highlight the elements that can be substantially linked to collective conscience rights. Arguments under this theme were constructed by organized collectives of healthcare professionals and representatives from non-participating institutions (proponents), alongside patient advocacy organizations, individuals who faced MAiD service obstructions, and lay citizens (opponents). We provide some illustrative quotes in Table 3.

In an important court challenge within the province of Ontario, some proponents focused on the rights of individual physicians to decline participation in MAiD—rights already protected by law but legally subject to certain limitations. Federal MAiD legislation necessitates effective referral when a healthcare professional with conscientious objections receives a MAiD request from a patient. Accordingly, in the Canadian province of Ontario, the College of Physicians and Surgeons of Ontario (CPSO) requires such physicians to refer a MAiD-seeking patient to an alternate healthcare provider. (There are similar requirements in other provinces.) Resisting this policy mandate, some Ontario physician groups and societies came together to file court petitions, arguing that their participation in the referral process makes them complicit in an act that conflicts with their conscience. Ultimately, the Ontario Court of Appeal upheld the CPSO policy, concluding that effective referral requirements may indeed violate some physicians’ conscience rights, but the harms suffered by patients would be much greater if the mandate were to be waived [59].

Although the case was concerned with individual rights and burdens, the appellants and intervening groups included religious alliances of healthcare professionals, patient advocacy organizations, and independent justice-based groups representing collectively held positions and interests. Proponents viewed the CPSO policy as an institutionalized infringement upon Charter-protected conscience rights for individuals [60,61,62]. Opponents representing patient rights were concerned that similar lobbies of other groups of conscientious objectors may form in future, seeking large-scale legal exemptions for those who do not wish to offer MAiD for reasons of conscience. (See for example, the federal Bill C-230 [63] and the provincial Bill 207 [64], neither of which passed.) Furthermore, some opponents highlighted an important discrepancy in legal protections, namely that unwilling healthcare professionals remain protected (with some legal limits) from having to perform MAiD at participating sites, but many MAiD-favouring professionals working at non-participating sites lack similarly protected rights to perform a procedure they personally support and deem medically appropriate (unless their patient transfers elsewhere) [65,66].

Notably, arguments within this theme engaged with the problem of patients needing to transfer if unable to receive MAiD at some sites. Some proponents, that is, representatives from non-participating sites, spoke to the media, expressing sympathy for the encumbrances faced by patients and intentions to facilitate smooth referrals and transfers, with a commitment towards mitigating any associated patient discomforts. However, in computing the risk of harms and burdens, these proponents also appeared hesitant to acknowledge the potential impacts of institutional abstinence on MAiD accessibility. They argued that patient transfers are less intimidating today considering telephonic/digital supports widely available for referrals [26,67], in addition to various external structures and processes (provincially organized) for coordinating transfers wherever needed [68]. On the other hand, opponents articulated the risk of harm by highlighting the stress that patients endure when compelled to seek MAiD outside the sites in which they reside or receive most of their care. Institutional policies prohibiting MAiD procedures on-site were described as aggravating service inaccessibility—especially for highly vulnerable patients at these locations, for example, for those too frail to transfer elsewhere, those with high pain management needs, or those at risk of losing capacity for MAiD consent mid-transfer [35,41,69,70]. Across transfer incident news articles, these patients were reported to have faced significant physical and psychological distress in moving between sites to receive an assisted death. Finally, access barriers were perceived to be more pronounced in rural areas, where the only available healthcare facility could be a faith-based one, potentially creating further hurdles to access and increasing the burden on MAiD-supportive healthcare sites [71].

### 3.4. Where Are the Gaps Being Felt in MAiD Service Implementation?

Commentators identified the needs not met—and sometimes created by—gaps in MAiD governance and service organization structures. These concerns drew attention to the evolution of MAiD as a legalized practice in Canada and the differences in its conceptualization and enactment, compared to other permissive jurisdictions across the world. In general, commentators across the spectrum of the debate—both collective conscience proponents as well as opponents—believed that government intervention in and further legal protection of diverse interests is necessary for resolving the new hurdles emerging in light of non-participating healthcare institutions. We provide some illustrative quotes in Table 4.

Many religiously affiliated healthcare institutions have longstanding agreements with provincial health authorities enabling faith-based care practices [73]. Several policy documents referenced these existing agreements [74,75,76,77,78]. Although non-participating institutions had choices with regard to MAiD service provisions [75,77,79], some policies noted that MAiD eligibility assessments would occur onsite [74], while some did not [75]. These inconsistencies indicate that not all sites have had the same negotiations or outcomes at this time, and it is not necessarily clear whether continuity of care can be maintained for all MAiD applicants at such sites, without undue stress.

Comparisons were drawn with countries like the Netherlands, where physicians had long been protected against legal prosecution as due-care criteria were developed in clinical practice—prior to the enactment of assisted dying legislation. It was argued that such jurisdictions had the opportunity to formulate legislation in response to circumstances as they evolved [34]. In contrast, the law in Canada was passed before testing such ground realities. Commentators noted that this deprived Canada of the crucial benefit of testing new standards of patient care, charting ethical conundrums through practice, and discerning the infrastructural limits of the health system in advance. With healthcare being a provincial responsibility in Canada, commentators further claimed that Bill C-14 (the federal government’s legislation on MAiD) concerned itself mostly with the rationale and criteria for determining MAiD eligibility, while leaving the responsibility of implementation and delivery to the provinces. Therefore, in the absence of prior MAiD-related experience and training, some commentators perceived provincial health authorities, professional regulatory bodies, and discrete healthcare organizations as struggling to adhere to radical and unfamiliar service regulations [42,80,81]. In this context, the province of Quebec was hailed as a possible exception, where medical training and public health education measures had commenced prior to the federal legislation [34]. 

In the province of British Columbia, the media reported cases where some healthcare institutions were portrayed as trying to employ dubious processes to restructure their governing boards to reach a MAiD-abstaining majority stance [81,82,83]. Similarly, there were reports of smaller standalone hospices being absorbed into larger faith-based healthcare organizations, under which MAiD would no longer be offered to the public. When such events occurred in remote or rural locations, collective conscience opponents expressed concern about the obstruction to MAiD access in these communities. In the province of Ontario, the reinstated Patients First Act (2016) [84] included what some opponents described as caveats slipped in covertly to strengthen institutional power to opt out of MAiD. This allegation referred to new legislative amendments to ensure that hospitals “shall not unjustifiably … require the board of a hospital that is associated with a religious organization to provide a service that is contrary to the religion related to the organization” [84]. Although this stipulation was designated at the provincial level, opponents were concerned that discrete healthcare institutions may add similar caveats to their own policies amidst widespread legislative ambiguity. This conflict was also seen in the province of Manitoba, where MAiD-abstaining sites were asked to ensure “that patients still have access to all aspects and processes associated with MAiD without delay” [85] (s5.10) and yet to communicate in writing that those deemed “too ill or too frail to be transferred” [85] (s5.15) may ultimately be unable to access MAiD.

## 4. Discussion

While the legalization of assisted dying was a major legislative landmark in Canadian medical history, support for MAiD remains far from universal and most certainly entangled with other legislative rights and freedoms. With conscientious objections being voiced by healthcare providers at the individual as well as collective levels, the variability in public views about MAiD and its potential impacts on patient care and service access are important considerations. In this context, institutional non-participation in MAiD presents unique challenges that have thus far been neglected in the literature. Given the absence of substantial systematic evidence on the issue, and the difficulty of gathering such evidence, we used data in the public domain to collate and characterize stakeholder perspectives, identifying the key themes of argumentation underpinning these.

### 4.1. Findings

The four principal themes that emerged in our analysis involved concerns with extending conscience rights to collective entities—especially, taxpayer-funded healthcare institutions; the perceived (in)congruity of MAiD within palliative medicine; the challenge of balancing competing stakeholder rights and burdens; and the inconsistencies in MAiD service organization and implementation.

Within the first theme, we found that commentators differed fundamentally in how they conceived the relationship between an institution and the people associated with it. This resulted in divergent views about whether conscience rights can be extended to institutions beyond the purview of individuals, or not. Proponents believed that institutions could claim legal protections for conscience rights because they were inseparable from the individuals who founded or operated them, while opponents did not share this view. In relation to this, it is worthwhile to consider some prior legislative evidence in Canada that has supported the notion of moral conscience for institutions as a whole. In the 2015 Loyola High School v Quebec case, the Supreme Court of Canada recognized the collective religious rights of institutions under section 2(a) of the Canadian Charter of Rights and Freedoms. However, the court ruling in the case stated that “[A]n organization meets the requirements for section 2(a) protection if (1) it is constituted primarily for religious purposes, and (2) its operation accords with these religious purposes” [86] (para 100). Legal experts have pointed out that healthcare facilities do not meet these criteria as they have not been set up to engage primarily in religious activities, and instead, their operations are focused on the delivery of medical services [87]. Overall, while there seems to be little legal basis for extending conscience rights to MAiD-opposed institutions, we found that stakeholders driving the debate continue to be divided on the subject, indicating a yet unmet need for consensus.

The second theme revealed that, despite MAiD services being integrated within publicly funded healthcare, there are disagreements about whether death-hastening procedures are compatible with the core palliative mission. Previously in the literature, reluctance towards incorporating assisted death within medical practice in general, and specifically within palliative care, has been related to the “paternalistic and death-denying” [88] (p. 194) attitudes of medical professionals, suggesting that the intentional hastening of death may be viewed as inherently opposed to medicine’s quest to heal and treat. Others have noted that the acceptance of one’s mortality, and the attempt to hasten death, may be perceived as a sign of suicidality [89]. Our findings suggested the persistence of these ideas in how palliative care organizations and professionals positioned their services and described their ethos, and occasionally, in news reports of staff reluctance towards administering MAiD inside palliative care wards (even at secular sites that offered MAiD) [90]. Although by no means universal, such sentiments may be indicative of the social stigmas that have lingered within public and clinical perceptions of death, especially pertaining to its occurrence within medicalized settings or contexts.

Captured within the third theme, we found that harmonizing the competing rights—of conscientious objectors and patients seeking MAiD—remains another unresolved issue, with commentators weighing the exercise of protected freedoms against the potential suffering or harms endured. In the (very few) news reports where patients were able to receive an assisted death at a MAiD-abstaining institution, the process was described as being arranged covertly by the patient or their family, with minute planning to avoid discovery, amidst fears of end-of-life wishes being obstructed if caught, and often, with unfavourable consequences for involved parties, including for healthcare professionals who agreed to facilitate MAiD at an abstaining site. This indicates the extent to which patients may go to meet their needs for moral agency, autonomy, and empowerment in making end-of-life decisions, which are linked to quality of life and satisfaction with care services [91]. Such incidents, although rare, occurred despite the presence of effective referral policies that should have enabled the patient to access MAiD elsewhere, suggesting the likelihood of many known (and unknown) circumstances under which referrals to other sites are either unfeasible or undesirable.

The fourth theme involved commentators’ views on the structures facilitating and integrating MAiD within public health service provisions. On all sides of the debate, there were calls for policy action and clearer guidance to address the issue of abstaining healthcare sites and resultant patient transfers, and more generally, to ensure consistency in communication and service protocol to help protect competing interests. Given the diverse sociocultural fabric of Canadian society, we stress that some patients may be at risk of having their end-of-life wishes declined due to moral beliefs that differ from those of their healthcare providers, leaving them no alternative but to transfer elsewhere. Besides increasing distress for patients and families during a time of heightened physical and psychological vulnerability, healthcare professionals involved in their care (both MAiD-favouring and MAiD-objecting healthcare professionals) may also be at greater risk of moral distress and emotional burnout. Prior evidence has suggested that the assurance of MAiD service availability is important to patients, regardless of its actual utilization; however, policymaking must account for divergent moral stances, with some degree of compromise achieved through consensus-building practices [92]. Overall, the situation presents an ethically complex scenario, in which we found that many commentators perceived a regulatory obligation on the part of the government and policymakers.

In the ongoing debate surrounding institutional conscientious objections to MAiD, two fundamental principles appear to clash: the principle of quality of life that emphasizes the importance of dignity, autonomy, and the mitigation of suffering (as defined by each individual patient) [93], and on the other hand, the principle of sanctity of life that professes the intrinsic value and inviolability of human life (which is more applicable to institutional policy and practice) [94]. In Canada—and other permissive jurisdictions—the prevailing stance on assisted dying appears to lean towards prioritizing quality of life over sanctity of life. Therefore, decisions relating to end-of-life choices tend to favour patient conceptions of well-being and the alleviation of suffering, even if the preservation of life is compromised. The emphasis on quality of life aligns with a utilitarian or consequentialist approach, where the consequences of an action hold significance in determining its morality [95,96]. In contrast, the principle of sanctity of life aligns with a deontological approach, which adheres to accepted moral standards and duties regardless of their outcomes [97] and is closely associated with theological schools of thought [98]. Moreover, from the standpoint of philosophical anthropology, the question of defining who a human being is comes into play when grappling with these principles [99]. The diverse views on the nature of human beings, their rights, the significance attributed to their life and care experiences, the changing scope of medical professional practices, and the role of legislation conjointly influence the positions taken by both proponents and opponents of MAiD. Overall, bioethical discourse about conscience in assisted dying issues—particularly on an institutional policy and service implementation scale—highlights unresolved conflicts of principle and involves philosophical questions about human identity and value.

### 4.2. Gaps and Potential for Future Research

For nearly 30 years, public opinion polls have consistently shown that most Canadians are in favour of MAiD [100]. Our study considered whether institutional non-participation in MAiD might affect service access and how this is perceived and characterized by various stakeholders. Taken as a whole, there is sufficient indication that further systematic inquiry is necessary to understand the nature and scope of effect on all stakeholders—including on healthcare users and providers. Unsurprisingly, given the nature of our dataset, the evidence tended to be informational, descriptive, and episodic in nature, without necessarily capturing the full range of people’s experiences, positions, and viewpoints. Public opinion polls to investigate how Canadians feel about this particular facet of MAiD service organization may be useful for evaluating national sentiment about MAiD-abstaining healthcare institutions.

At present, several voices likely remain unheard, or at least, inadequately captured—including those of many patients who were not able to transfer at all, those who transferred smoothly without facing major encumbrances, and healthcare staff (at both participating and non-participating locations) who assisted patients between sites. There is also little to no data on how socioeconomic status and sociodemographic differences might influence healthcare users’ abilities to navigate situations where a site declines to provide MAiD and an alternative location must be sought [101,102]. Moreover, since MAiD is a well-planned event and process, a large proportion of service users are likely able (and prefer) to have the procedure performed in their own homes, easing the burden on transferring and receiving sites. However, the fewer the number of patients affected adversely, the more likely it remains that the issue may escape research and policy attention, with impacted minorities left with little recourse in times to come. Therefore, it is necessary to determine how widely and deeply institutional abstinence from MAiD affects equitable access “on the ground” and to what degree the negative impacts (or the lack thereof) are mitigated by and reliant upon MAiD utilization in home-based settings. From a normative standpoint, it is also crucial to ponder whether the presence of alternative MAiD access routes and fewer cases of MAiD access obstructions can fully justify scenarios where healthcare institutions refuse to offer publicly funded healthcare services. Public deliberation about how these challenges can be resolved through acceptable compromise have been suggested as a potential solution worth examining [92].

Since the administration of MAiD procedures requires patient consent to be obtained explicitly and confirmed repeatedly, thorough, ongoing, and unbiased discussions about EoLC decisions must take place in a safe, non-judgmental environment between patients and staff. We contend that, in some cases, holding such discussions may become challenging when on-site staff—with whom patients are likely to be most familiar—are not the ones to review MAiD options with them as part of medical care planning. In many cases, patients may consider some types of facilities to be their homes (e.g., long-term care homes) or, at least, places of familiarity and routine, where they have established social connections with others and formed trusting relationships with healthcare staff. Some situations where patients (and families) must find and move to alternate care sites can be reasonably expected to exacerbate their physical and emotional suffering. To our knowledge, no studies have been conducted to explore the experiences of patients who request MAiD at such sites under these circumstances, highlighting a key gap that must be addressed to understand the contours of this ethical dilemma.

Finally, as some commentators have mentioned, there may be valuable lessons to learn about addressing challenges in service regulation from other permissive jurisdictions across the world [103]. For example, the Belgian media has covered incidents where religiously affiliated healthcare institutions were reportedly faced with losing their licences or fined for refusing medical aid in dying. By and large, with widespread public support for assisted dying in Belgium, resistance to the practice has been limited, and many religious institutions have adjusted their ethics codes to align with the law [104]. However, in May 2020, after an investigation by the Vatican and numerous consultations between the concerned parties, 15 Belgian centres affiliated with The Brothers of Charity were stripped of their Catholic status for allowing assisted deaths on site. While Belgian society, much like the Canadian public, is largely supportive of assisted dying, its policies on the matter have been criticized for being unsecular. With some commentators similarly describing Canada’s stance as totalitarian and intolerant (towards those with religious reservations about MAiD) [32,50], future research and policy on the subject should investigate the complexities presented by the complex sociocultural composition of Canadian society.

### 4.3. Limitations

Before concluding, we will note some limitations of this study. This paper reviews the debate on institutional objections to MAiD as it continues to progress within the public domain in Canada. Given the nature of our dataset, the nuances of various stakeholder interests and views may not have been captured in full in our analysis. Moreover, certain stakeholder groups may not have engaged with public platforms as much as others—or at all—making it possible that the key argument themes we have outlined above would shift with their inclusion (or increased activity) across these platforms. Secondly, much of our dataset comprises articles that interpret stakeholder stances through a journalistic lens; that is, they were crafted by authors situated outside or not directly engaged with or personally affected by issues of institutional MAiD abstinence and resultant patient transfers. These biases in opinion and discrepancies in stakeholder representation, in fact, reflect the current state of publicly accessible narratives and information on the subject. Therefore, we draw no definitive conclusions about, or from, stakeholder accounts and positions presented herein. Rather, we describe how these materials construct the subject of institutional objections to and non-participation in MAiD in Canada today, making the case for further systematic inquiry.

## 5. Conclusions

Scholars from the sociology of death and dying have long described death as a subject located outside the realm of mainstream social life [105,106]. Federal legislation on assisted dying in Canada and ensuing policy changes to implement MAiD across provinces have thrown into the spotlight the flux of values pertaining to death and dying within Canadian society. Divergent citizen views about and experiences with institutional resistance to MAiD reflect dissonant social realities as well as challenges in integrating EoLC, particularly MAiD, within the health system. There are other important questions to consider about what this means for the state of democracy in Canada, where the law must, on the one hand, strive to ensure reasonable equity in healthcare access, and, on the other, safeguard universal freedom of religion and protect conscience rights. In the absence of an official centralized source, public knowledge about MAiD-abstaining institutions, patient transfer incidents, and policy developments on the subject is derived primarily from the media and organizational information sources. Other avenues for Canadians to consume, produce, or engage with stakeholder accounts or to obtain local institutional policies remain limited.

In our analysis, it appeared that various stakeholders, regardless of their stances, often utilized public platforms for announcing, reaffirming, or revising their positions on the issue of institutional MAiD oppositions. First, this implies that there may be limited opportunities for direct exchange between stakeholders for reaching consensus over the contentious aspects of MAiD policy and practice. Second, public narratives may determine how Canadians inform themselves of their healthcare rights pertaining to MAiD accessibility, and more specifically, how Canadians gauge the ability of the healthcare system to accommodate their wishes, should they wish to avail themselves of MAiD. Third, stakeholder arguments, as constructed across public platforms, have the potential to (re)shape how Canadians perceive the attitudes of palliative care providers within their local communities and, importantly, how people’s personal moralities may shift with respect to recurrent arguments across publicly accessible domains. Overall, public domain materials constitute an important territory within which moralities—and practicalities—surrounding MAiD implementation can be located and traced as they unfold in post-MAiD Canada. On rare occasions, institutional non-participation results in patient transfers, and while such incidents do not signify the majority of MAiD request outcomes across the country, the issue should not remain overlooked. Instead, in future years, the subject should receive robust scholarly attention to understand its context and significance within the goal of universally available and accessible EoLC services.

## Figures and Tables

**Table 1:** Theme 1—Illustrative examples.

WHO HAS THE RIGHT TO CONSCIENCE?
**Collective/Institutional Conscience Proponents**
“Anyone who comes here [abstaining site] knows what our policy is. And if they don’t like the policy, they should go somewhere else” [23].—*Representative of nursing home ethics board, speaking to The Globe and Mail,* 18 January 2018
“Is it not hypocritical to say, ‘Let us use the hospital you built on the cornerstone of your faith, but when we use it, let us force you to remove that cornerstone?’ Surely, the institution would then be lacking its essential cornerstone. It is a bit like a man who moves with his family into a gracious friend’s house. When the man arrives, he insists his friend’s family move out at once to make room for his own relatives, ignoring the fact that the house was built in the first place for his friend’s family” [24].—*Lawyer, authoring an editorial in the Ottawa Citizen, 24 October 2016*
“What is a brick-and-mortar institution? Faith-based health care exists because of the people who founded it and work in it. If there was some sort of societal calamity in which the hospital building was no longer usable, what would happen to faith-based health care? It would probably revert to a field hospital in a tent or some such thing, with the same mission to patients […] There is a human reality to these institutions that have served people regardless of race or belief, and the new lay structures are still doing the same” [25].—*Archbishop of Roman Catholic organization, authoring an editorial in the Winnipeg Free Press, 8 July 2017*
**Collective/Institutional Conscience Opponents**
“…when it’s combined with the fact that the person, by virtue of [institutional] policy, is being forced to stay in a place where they are being denied something that the Supreme Court says they have a right to because of religious issues that shouldn’t be determining their health care… potentially, I think you do have a Charter issue” [26].—*Legal expert, speaking to the Winnipeg Free Press, 24 February 2018*
“A publicly funded hospital takes all patients, regardless of religious affiliation (or lack thereof). The hospital is not delivering ‘Catholic care’, it is delivering medical care that is nondenominational, non-religious and independent of religious oversight. Priests do not determine the care in Catholic hospitals, physicians and other healthcare professionals do. While an individual physician may have a Charter-protected religious right to ask another doctor to take over the role of ending a life, a hospital has no constitutional right to prohibit all of its physicians from doing so. Hospitals have no conscience, only the people who work in them do” [27].—*Professor of health law and ethics, authoring an editorial in the Times Colonist, 18 October 2016*
“But what if the faith or moral position of one of these religions suggested that women receive lesser or different standards of treatments than men? Or if their religion didn’t allow them to provide certain services to homosexuals or members of the LGBTQ community? What then? I suspect the government’s reaction wouldn’t be quite so accommodating […] The idea of forcing patients in acute distress to move to another hospital if they want to even discuss euthanasia with a doctor is, frankly, cruel and inhumane” [28].—*Policy analyst and digital media specialist, authoring an editorial in the Winnipeg Free Press, 29 November 2016*

**Table 2:** Theme 2—Illustrative examples.

CAN MAiD BE CONSIDERED A PALLIATIVE PRACTICE?
**Collective/Institutional Conscience Proponents**
“Our goal is to help people live to the end of their natural lives. When people get good hospice and palliative care, the desire for assisted death disappears […] In other jurisdictions, people will seek assisted death as Plan B. They won’t go there if they get good palliative care” [37].—*Representative of hospice palliative care organization, speaking to the Ottawa Citizen, 8 October 2016*
“…a while back, Manitoba’s College of Physicians and Surgeons asked for public feedback on physician-assisted killing but called it ‘physician-assisted dying’. Physician. Assisted. Dying. Of course, we all want medical assistance when we die: we all want clean bandages, food, and morphine as death takes its course. But by ‘physician-assisted dying’, our College of Physicians and Surgeons means to kill us, not care for us. This. Is. Orwellian” [38].—*Member of the public, in a letter to the editor of the Winnipeg Sun, 15 December 2016*
“…at the most fundamental level, [MAiD] contradicts the basic tenets of Catholic health care—wherein life is held to be sacred from conception to natural death—and not permitted in Catholic health care institutions” [36].—*Providence Health Care memo to clinical leadership team and medical advisory committee, as quoted in The Globe and Mail, 25 February 2016*
“The core issue is that Catholic and faith-based organizations are committed to the inherent dignity of every human life and would never intentionally hasten the end of life” [23].—*Representative of Providence Health Care, speaking to The Globe and Mail, 8 January 2018*
“Canadian healthcare professionals must be free to fulfil their calling to care for, and not to kill, those who are sick and dying” [39].—*Professor of law and professor of nursing, co-authoring an editorial in the National Post, 11 February 2021*
Collective/Institutional Conscience Opponents
“How can a doctor turn a deaf ear to the pleas of someone dying from metastatic cancer, who has only a few days to live? And some of these same physicians claim their decision is based on religious beliefs!” [40].—*Physician, authoring an editorial in the Prince Albert Daily Herald, 20 December 2016*
“It is, in effect, telling your patient ‘tough luck’. The most vulnerable patients will lose their access to MAiD if they’re unable to be transferred. That’s a pretty heavy price to be paid by a patient who is with a grievous medical condition, who’s suffering intolerably” [41].—*Professor of ethics, speaking to the Winnipeg Free Press, 2 January 2018*
“In the early days of this, we got hate mail. I’ve been publicly identified early on as doing this work. We have an email address, and I’ve been told I’m a murderer and other things […] We could become targets, and we don’t want that to happen” [42].—*MAiD provider and policymaker, speaking to the Toronto Star, 17 July 2016*
“Carting the very sick backwards and forwards for [MAiD eligibility] assessments, or worse still, keeping them alive against their well-considered wishes, hardly accords with that [do-no-harm] dictum” [43].*—Physician and patient advocate, authoring an editorial in the Lethbridge Herald, 10 May 2017*

**Table 3:** Theme 3—Illustrative examples.

ARE THERE IMBALANCES ACROSS DIVERSE STAKEHOLDER RIGHTS AND BURDENS?
**Collective/Institutional Conscience Proponents**
“This isn’t black and white. You can’t force someone to provide a sensitive service at a time that is so critical to people when they are diametrically opposed to it” [36].—*Former provincial health minister, speaking to The Globe and Mail, 25 February 2016*
“We also feel that people do have a right to information […] We have no problem with providing that information, but there’s something different about a direct referral, that actually says that you need to find someone who will carry through on what we see as a very harmful action. Do you want to force doctors to have to harm people that they care for? And many of these doctors do see this as a harmful action” [55].—*Representative of Roman Catholic organization, speaking to the Moose Jaw Times Herald, 22 June 2016*
“Some assisted dying opponents feel their principles have made them targets. Quebec’s palliative care centres unanimously decided last fall to refuse to provide assisted dying because of their philosophical objections. They are now fearful of losing their public funding. I know of people who were trying to start a new palliative care centre that they’d been working on for several years and were told this year that they would not get public funding… Is that because they don’t intend to euthanize patients? I don’t know but it could be” [42].—*Physician, representative of the Physicians’ Alliance Against Euthanasia, speaking to the Toronto Star, 17 July 2016*
**Collective/Institutional Conscience Opponents**
“The worst-case scenario is we would go along the 401 [highway], quite frankly, to see if we could find a partner [health facility] that would support the patient. It illustrates the real problem for patients in facilities that get a free pass on medical assistance in dying. They are treated like a hot potato” [56].—*Representative of patient advocacy organization, speaking to the Windsor Star, 18 February 2017*
“We say we’re trying to balance competing human rights, the rights of the Catholic Church and the rights of patients. But there’s little balance when a hospital’s values trump the best interests of a patient, when a dying man’s dignity is sacrificed on the altar of someone else’s religion” [57].—*Independent senator, Senate of Canada, authoring a column in the Edmonton Journal, 29 September 2016*
“…the question, therefore, is not whether the Catholic Church’s stance is justifiable, but whether they should be allowed to impose that stance upon non-supporters through their participation in health care” [58].—*Member of the public in a letter to the editor of the Winnipeg Free Press, 13 July 2017*
“Who is medical assistance in dying for, if not people who are incredibly ill and often incredibly frail? […] What we see in Manitoba, with this appalling hodge-podge approach in these long-term care facilities, is incredibly obstructionist” [41].—*Representative of patient advocacy organization, speaking to the Winnipeg Free Press, 2 January 2018*

**Table 4:** Theme 4—Illustrative examples.

WHERE ARE THE GAPS BEING FELT IN MAID SERVICE IMPLEMENTATION?
**Collective/Institutional Conscience Proponents**
“In every jurisdiction in the world, conscientious objection is recognized in some form […] The only governments in the history of humanity that have stripped away the conscience rights in this way are totalitarian governments. Are we going to get to the point where there’s an ethics test at the beginning of medical school, and if you have too much in the way of ethics, you’re going to be screened out?” [32].—*Journalist, authoring a column for the Sun Times, 23 September 2016*
“We as organizations would then be placed in the position of determining whether we abide by a regulation or whether we abide by the conscience and collection voice of our denominations […] The government can certainly impose upon facilities its will, but then the individual denominations would then need to determine whether they would continue to operate those facilities” [55].—*Representative of religious charity organization, speaking to the Moose Jaw Times Herald, 22 June 2016*
“These are big questions of public policy and private morality. How can we force the province’s 21 publicly funded Catholic hospitals to do what they know they cannot? Cutting off government money would surely be a Pyrrhic victory and morality play, not to mention a false economy. For it is no accident that faith institutions are among the most motivated—and irreplaceable—providers of palliative care […] Equally, some Ontario doctors are in a quandary because the College of Physicians and Surgeons, which regulates their practice, has ruled that if they refuse to act on a MAiD request, they must provide a referral to another practitioner who will. This seems an abuse of authority. No right is absolute and matters of conscience should not be arbitrarily circumscribed if reasonable compromise and accommodation is possible […] Coercion is a solution in search of a problem, a dead end given that we have other pathways to get people where they want to go to die” [68].—*Journalist, authoring a column in the Thunder Bay Chronicle Journal, 14 April 2017*
**Collective/Institutional Conscience Opponents**
“The need for government to re-engage with doctors is essential if improving patient care is truly one of their priorities” [72].—*Representative of provincial medical association in a press release, as quoted in Canada NewsWire, 29 March 2017*
“Some of the language and the contradictions in here really gave me pause. They say they’re directing the health authorities and objecting facilities to develop policies to ensure that patients aren’t delayed or blocked from MAiD but then a line later admit that the ban on MAiD at certain facilities makes that literally impossible in some cases. The circle just cannot be squared here” [41].—*Representative of patient advocacy organization, speaking to the Winnipeg Free Press, 2 January 2018*
“This is the cruellest hospital policy that I have ever encountered in over 30 years of medical practice” [35].—*Physician, in resignation letter to hospital board, referring to the policy of non-participation in MAiD, as quoted in the Times Colonist, 19 October 2016*
“The viability of ethical objections hinges on effective referrals. The healthcare system can’t function if every doctor, nurse and pharmacist can […] withhold services to which patients are legally entitled. It’s what separates conscientious objectors—who acknowledge patients’ right but can’t in good conscience participate—from mere moralizers, who would impose their own values to restrict other people’s choices” [51].—*Journalist, authoring a column in Northern News, 3 October 2016*

## Data Availability

The data presented in this study are available on request.
